# Smartphones for Real-time Assessment of Adherence Behavior and Symptom Exacerbation for High-Risk Youth with Asthma: Pilot Study

**DOI:** 10.2196/pediatrics.9796

**Published:** 2018-10-05

**Authors:** Ronald John Teufel II, Sachin K Patel, Anita B Shuler, Anne L Andrews, Michelle Nichols, Myla D Ebeling, Erin Dawley, Martina Mueller, Kenneth J Ruggiero, Frank A Treiber

**Affiliations:** 1 Division of General Pediatrics Department of Pediatrics Medical University of South Carolina Charleston, SC United States; 2 Technology Applications Center for Healthful Lifestyles College of Nursing Medical University of South Carolina Charleston, SC United States; 3 Children's Hospital Medical University of South Carolina Charleston, SC United States; 4 College of Nursing Medical University of South Carolina Charleston, SC United States; 5 Division of Neonatology Department of Pediatrics Medical University of South Carolina Charleston, SC United States; 6 Department of Psychiatry Medical University of South Carolina Charleston, SC United States

**Keywords:** children, ecological momentary assessment, medication adherence, medication monitoring device, mHealth, mobile phone, symptoms, youth

## Abstract

**Background:**

Youth with asthma who have poor medication adherence, have limited access to care, and are frequently seen in the acute care setting are often termed “high risk.”

**Objective:**

This study aimed to design and test the feasibility of using smartphone technology to assess contextual factors that may impact changes in daily medication adherence and to identify new symptom episodes among high-risk youth with asthma in their home environment.

**Methods:**

Youth aged 8-17 years with high-risk asthma from 2 children’s hospitals were eligible for the 2-month study. An app was downloaded on participants’ phones at enrollment. Daily text message (short message service) reminders were sent to complete ecological momentary assessment of asthma symptoms and other contextual factors such as emotional state using the app. Bluetooth inhaler devices were used to record timestamps of inhaler use with the ability to review and manually enter data. The acceptability was assessed with surveys, key informant interviews (KII), and frequency of days with asthma data. KII data were used in an iterative design approach to identify challenges, strengths, and suggestions for maximizing use. Generalized linear mixed modeling was used to preliminarily explore contextual factors associated with changes in daily adherence.

**Results:**

We enrolled 14 children aged 8-16 years (13/14, 93% were African Americans). Over the 2-month study period, participants reported coughing (42/110, 38%), wheezing (8/111, 7%), chest tightness (9/109, 8%), boredom (57/109, 52%), and 10 new asthma symptom episodes. The controller medication adherence was 30%, which increased significantly on days with asthma symptoms or boredom. Data were received on 89% (606/681) of study days. Surveys and KIIs suggest acceptability among youth and their caregivers. Challenges reported during the study included lost or damaged phones and available memory.

**Conclusions:**

Youth and their caregivers reported the acceptability of using smartphones for real-time asthma monitoring. Overall, the controller medication adherence was low but increased significantly on days with reported asthma symptoms or boredom, suggesting that daily contextual factors may be associated with a change in the adherence behavior.

## Introduction

Asthma impacts the lives of 7 million children in the United States costing US $9 billion annually with emergency department and hospital admissions accounting for half of these costs [1-4]. Children with frequent emergency department and hospital visits are often termed “high risk” and more likely to be from a minority race or ethnicity and experience unique barriers such as decreased access to care and more severe disease [5]. Uncontrolled asthma and frequent exacerbations not only negatively impact health but also the quality of life and school attendance, and contribute to missed parental work days [6,7]. Effective preventive care strategies exist to control asthma and prevent its negative outcomes. However, these treatments are seldom delivered with satisfactory adherence among high-risk youth with asthma [8]. Medication adherence among children is further complicated as responsibility for controller inhaler medication delivery gradually transitions from parents to youth. Less than 20% of children aged 7 years are responsible for asthma medication self-management compared with 100% of 19-year-olds’ with asthma [9].

Barriers to controller medication adherence among youth with asthma include forgetfulness, poor asthma knowledge and false beliefs, child avoidance of inhaler use at school, and parental stress with medication delivery [10-14]. Less is known about other social and emotional contextual factors (eg, anger, boredom, and symptoms), and how changes in these factors over time impact the day-to-day adherence behavior for youth with asthma. Emotional factors, such as anger or panic, are frequently reported in association with asthma symptoms, but their effect on asthma self-management including adherence is poorly understood [15,16]. Daily experiences such as mood and motivation have been shown to impact medication adherence in other disease states [17]. Previous studies examining effects of contextual factors on the asthma medication adherence have been based on retrospective surveys, self-reported adherence, and asthma diaries, which are time-consuming, potentially biased, and costly. The adoption of smartphones may offer a solution.

Nearly 75% of US youth have or have access to a smartphone, 92% report going online daily with any mobile device, and 24% report going online “almost constantly” [18]. Ecological momentary assessment (EMA) is an established technique developed within behavioral sciences that enables researchers to monitor disease symptoms, emotional state, and other contextual factors frequently in a real-time natural environment avoiding recall bias [19,20]. The patient-reported inhaler use collected through the EMA has been correlated with validated severity self-report measures (eg, Asthma Control Test) and used in clinical trials with smartphone-based interventions [20,21]. Medication adherence devices previously used in clinical trials are now emerging as a practical tool for clinical practice as they readily connect with smartphones via Bluetooth technology, avoiding inaccuracies associated with self-report [22]. This emerging adherence technology combined with the near-universal adoption of smartphones enables real-time assessments of medication use and patient symptoms and mood, reducing the required effort and cost of attaining real-time assessments of youth with asthma, which may facilitate a better understanding of factors influencing daily changes in adherence behavior [23].

This study aimed to design and pilot-test the feasibility of the Smartphone Asthma Monitoring System (SAMS) that utilizes Bluetooth inhaler monitoring and EMA of contextual factors for youth with high-risk asthma. We used an iterative design approach to maximize participant acceptability among high-risk youth with asthma and their families to address specific barriers to using the technology. Secondarily, this study aimed to explore contextual factors that may impact daily adherence behavior (eg, anger, boredom, or asthma symptoms), as well as understand the utility of the collected data for the identification of newly symptomatic patients.

## Methods

### Study Population

Youth aged 8-17 years were eligible for participation in this 2-month study. The inclusion criteria were as follows: (1) high-risk asthma, defined as patient having emergency department or hospital visit for asthma in the previous 12 months or the child’s primary asthma provider in the outpatient setting having the opinion that the youth is high risk for future asthma visits to the emergency department or hospital owing to concern for noncompliance or poorly controlled asthma; (2) prescribed a controller and rescue medication compatible with Bluetooth inhaler cap; (3) caregiver or youth own and are able to use a smartphone compatible with Bluetooth devices; (4) English speaking; (5) identified primary care provider with at least one visit in the last year; and (6) ≥1 identified caregiver present for enrollment. This study was approved by the Institutional Review Board at the Medical University of South Carolina.

### mHealth Platform

#### Overview

**Figure 1:**
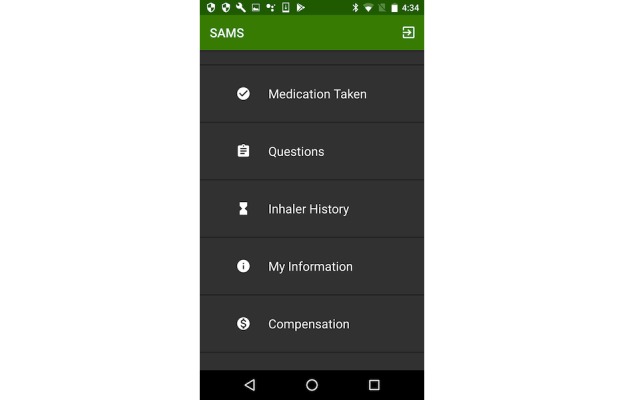
Screenshot of the Android smartphone app home screen. Image Source: Ronald J Teufel II.

The SAMS team used an iterative design process with frequent youth, caregiver, and provider engagement to develop a secure mHealth platform. The platform includes a smartphone app (Android operating system) and Bluetooth inhaler caps for enrollees and a Web-based portal for providers or investigators. The app serves the following 4 major functions: (1) manual medication use entry; (2) EMA questionnaire completion; (3) access to inhaler use history; and (4) tracking study compensation (Figure 1).

**Table 1:** Reported asthma symptoms and mood association with changes in daily adherence.

Ecological momentary assessment	Change in adherence^a^			
	Not at all	A little	A lot	Extremely
Are you coughing?	Reference	+6.8% (−3.3% to 16.9%)	+10.5% (−19.1% to 40.1%)	N/A^b^
Are you wheezing?	Reference	+1.0% (−15.0% to 17%)	N/A	N/A
Does your chest feel tight?	Reference	+8.0% (−7.4% to 23.5%)	N/A	N/A
Are you stressed?	Reference	+4.1% (−10% to 18.2%)	N/A	−1.7% (−42.4% to 39.1%)
Are you angry?	Reference	+7.3 % (−9.2% to 23.7%)	+11.1% (−30.0% to 52.3%)	+7.4% (−16.9% to 31.7%)
Are you bored?	Reference	+2.4 (−7.3 to 12.1)	+11.3 (−16.8% to 39.3%)	+20.3 (2.4% to 38.2%)^c^
Are you happy?	+4.5% (−15.2% to 24.1%)	−2.6% (−15.8% to 10.7%)	+3.1% (−6.5% to 12.7%)	Reference
Are you relaxed?	+3.9% (−12.5% to 20.3%)	+0.4 (−14.9% to 15.7%)	−0.8% (−11.1% to 9.4%)	Reference

^a^Estimates were obtained from generalized linear mixed modeling accounting for the longitudinal nature of data, with patients included as random effect.

^b^N/A: Not applicable.

^c^*P*=.03.

##### Ecological Momentary Assessments

Our team of asthma providers and behavioral scientists developed EMA questions to assess youth symptoms (eg, coughing, wheezing, and chest feeling tight) and contextual factors, such as emotional state (eg, stressed, angry, bored, happy, and relaxed), relevant to asthma or adherence behavior. To help ensure question clarity, we piloted EMA questions with 3 youth aged 8-15 years and their parents in a small group session before study recruitment. The app was designed to enable youth and caregivers to answer EMA questions securely through their smartphone with minimal effort by clicking the “Questions” tab within the app. Table 1 and Multimedia Appendix 1 list the specific questions including the 4-point Likert scale.

##### Bluetooth Inhaler Caps

At enrollment, Bluetooth inhaler caps (CareTRX, Cambridge, MA) were attached to both rescue and controller inhalers, paired with the child’s or caregiver’s smartphone, and successful transfer of data was assessed. Enrollees were instructed to use the inhalers with the cap attached and transfer the cap if a new inhaler was obtained. The caps can store up to 400 inhaler use events. Once the smartphone and cap were within the Bluetooth range (approximately 15-20 feet), the inhaler use data automatically downloaded to the phone. The timestamps were immediately transferred to the Web-based portal and were viewable within the SAMS app in the “Inhaler History” tab. Participants were permitted to enter controller or rescue medication use manually (under the tab “Medication taken”) to address situations in which Bluetooth caps were not communicating with the smartphone or in the event of other barriers to data transfer.

#### Web-Based Portal for Providers

A Web-based portal was designed for providers to document and store enrollment information (eg, email, telephone number, and study identification number) and enable review of youth asthma data in real time. The app securely transferred all data to the Web-based portal on an encrypted server. The portal has the capability to automatically process data and send preprogrammed reminders (eg, inhaler use or study protocol reminders) or reports (eg, adherence or symptoms reports) to patients, caregivers, or providers through email, smartphone reminders, or short message service (SMS) text messages.

### Study Design

#### Enrollment and Procedures

Recruitment occurred from 2 university children’s hospitals and affiliated ambulatory clinics (Medical University of South Carolina and University of South Carolina or Palmetto Health) between August 2015 and November 2016. Referrals were received from physicians, respiratory therapists, nurse practitioners, and pharmacists performing inpatient medication reconciliation. After informed consent was obtained, youth and parents were instructed on how to download the SAMS app, including software for Bluetooth inhaler caps. Children and parents received a demonstration of the SAMS app and Bluetooth inhaler caps. Study personnel documented age, race, ethnicity, comorbid diagnosis, and medications based on caregiver report and provider notes. All participants were instructed to enter EMAs daily. Participants received daily SMS text message reminders to complete their 8-item EMA questionnaire. Participants did not receive reminders to take their asthma medications.

Participants returned for a final study visit including a usability survey and key informant interviews (KII) after the 2-month study period. Parents of the enrolled youth were given gift cards of US $50 at enrollment and US $150 at the final study visit to compensate for their time, travel, and any additional cell phone charges accrued during the study.

##### Usability Survey

The usability survey was designed based off the concepts of ease of use, and intention to use from the technology acceptability model [24]. The survey was modified from previous technology development projects and used a 5-point Likert scale (strongly disagree to strongly agree) [25]. We also included questions regarding comfort with monitoring their disease by a smartphone based on our clinical asthma providers’ early feedback that youth and families may not feel comfortable with providers knowing details on medication use.

##### Key Informant Interviews

Semistructured exit KII were conducted with participants (youth and parents) to better understand their experience with SAMS and how the design could be improved. Interviews were audiorecorded, transcribed, deidentified, and reviewed by our team to identify elements that could be redesigned to improve the ease of use. A more extensive qualitative analysis considering behavioral theory (eg, self-determination theory) will be reported separately.

#### Changes to mHealth Platform Through Iterative Design

**Figure 2:**
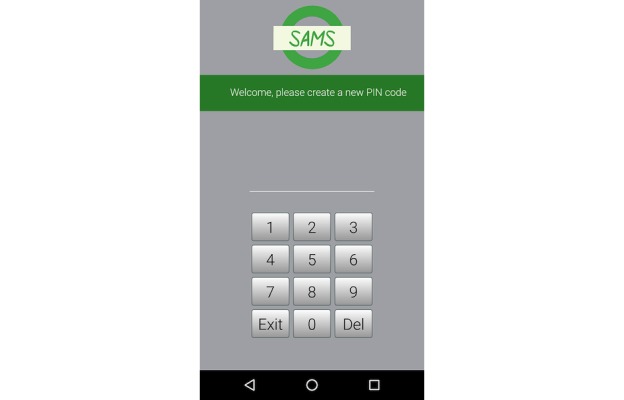
Screenshots of an Android smartphone app for youth and caregivers, including 4-digit log-in. Image Source: Ronald J Teufel II.

Recruitment was suspended at the midpoint of the pilot study (ie, after enrollee #7) to implement the identified redesigned elements. The redesign included a 4-digit auto-log-in (Figure 2), individualized SMS text message reminders and offer of technical support when no EMA data were reported over any 3-day period, and SMS text message delivery at enrollment and upon request with a link to reload the app on replacement phones.

### Outcomes

#### Medication Use and Adherence

Adherence to the controller medication (inhaled corticosteroids) regimen was calculated daily in 12-hour increments (12:00 am to 11:59 am and 12:00 pm to 11:59 pm) as the number of doses prescribed compared with the number taken over 12 hours to avoid the impact of medication overdosing and dumping [26,27]. The study group’s adherence was calculated by averaging daily adherence. Duplicative data (from Bluetooth cap and manual entry) were excluded at the time of data analysis if the device subsequently paired with the smartphone and transferred data.

#### Assessment of Asthma Symptom Day and New Asthma Symptom Episodes

Any day with reported asthma symptoms (eg, cough, wheezing, or chest tightness) was considered an asthma symptom day for that enrollee. Asthma symptoms days reported through the EMA immediately following enrollment were considered part of an initial asthma exacerbation. To test the feasibility of using EMA to detect new or recurrent asthma symptom episodes for early intervention, we developed a definition of new asthma symptom episodes to be applied to EMA data over the 2-month study period.

New asthma symptom episodes required (1) reporting asthma symptoms a minimum of 7 days postenrollment and (2) at least one EMA reported without asthma symptoms in the interim period. A participant was permitted to have >1 new asthma symptom episode during the 2-month study period, but the events had to be separated by a report with no asthma symptoms.

#### Emergency Department and Hospital Visits

A statewide clinical data warehouse developed and maintained by Health Sciences South Carolina was used to collect information on asthma-related emergency department and hospital visits for study participants. The data warehouse contains over 2.7 million unique patients encountered in the inpatient, outpatient, and emergency department from 11 member institutions, including both enrollment sites for this study [28]. Visits were reported in categories of 2- and 6-month pre- and postenrollment.

#### Data Acquisition as Outcome

This pilot feasibility study was performed to engage participants (youth and caregivers) in the design of a mobile technology approach to facilitate frequent information exchange about inhaler use and asthma symptoms with a clinical provider. The frequency of days with data acquired on inhaler use and asthma symptoms was viewed as an indicator of both the usability by youth and their parents and feasibility.

### Statistical Analysis

Our analysis included descriptive statistics for demographics, comorbid diagnosis, medications, the usability survey results, and percentage of days with asthma data acquired (EMA data or any asthma data including inhaler use or EMA). We analyzed the percent of days with data acquired before and after the app redesign using *t* test. We further investigated differences in the EMA reporting frequency by age, gender, and weekend versus weekday reporting with Pearson correlation coefficients or *t* tests. We used generalized linear mixed models with daily reports of asthma symptoms and emotional state predicting the outcome of daily adherence to controller medication accounting for the random effect of individual participants to explore whether the adherence behavior may be associated with individual EMA responses [17,23]. The models included a time variable for each study day to account for the longitudinal nature of our data with repeated measures of symptoms and EMA responses over time. This analysis enabled more accurate correlation between daily factors as it accounted for clustering on the patient level (eg, individual enrollee lower or higher adherence). Encounters for asthma in the emergency department or hospital during the 2-month study period were explored with participant-level data on reported asthma symptoms to determine whether preceding signals exist for an acute care encounter. Statistical analyses were performed using SAS version 9.4 (SAS Institute, Cary, NC, USA, 2013).

## Results

### Study Population

In this study, we enrolled 14 youth aged 8-16 years and their parents (Table 2). Of the 20 patients screened for enrollment, 14 (70%) participated in this study. While 4 patients screened did not meet the inclusion criteria (age <8 years, not on a controller medication, uncertain access to cellular service because of leaving country of residence for the majority of study period, or noncompatible smartphone), 1 participant consented but medication changes immediately after consenting no longer met the inclusion criteria. Of patients who were referred and met the inclusion criteria, only 1 declined participation citing they preferred to avoid research studies. Partial EMA or adherences data were used for 3 patients because of smartphones being reported as lost or damaged.

### Demographics and Clinical Characteristics

Among 14 participants, 13 were defined as high-risk asthma because of emergency department or hospital visit within past 12 months, and 1 was included because of the consideration of high risk by the primary provider. Overall, 14 patients reported comorbid diagnoses, including attention deficit hyperactive disorder (3/14), allergic rhinitis (2/14), eczema (3/14), food allergies (1/14), gastroesophageal reflux disease (1/14), obstructive sleep apnea (1/14), and vasovagal syncope (1/14). All patients were receiving both controller and rescue medications. Additional medications at the time of enrollment included antihistamines (5/14), nasal steroids (3/14), leukotriene receptor antagonists (1/14), proton-pump inhibitors or H_2_ blockers (2/14), oral corticosteroids (4/14 total with 3/14 having a 3- to 5-day course of oral prednisone and 1/14 oral dexamethasone), stimulant medications (2/14), and Omalizumab (1/14). The overall controller medication adherence was 30%.

### Youth and Caregiver Usability and Response to Redesign

Multimedia Appendix 2 presents usability survey responses. All youth and parents agreed or strongly agreed with the statement “I feel comfortable with a doctor or a nurse monitoring my health information using mobile technology.” During the exit interview, 1 parent stated, “I had no challenges, at all” “it was easy, and I’m not technology savvy.” Another parent highlighted the benefits of smartphone use over using computers through her statement of “...a lot of people, with the phone, they carry that everywhere. With your computer, most of the time you’ve gotta wait ‘til you get home and check it’.” Previous studies have also demonstrated African American adolescents, and their parents prefer technology interventions that are not based on a home computer platform [21]. Our iterative design process used enrollee feedback to inform the redesign of our technology-based approach for enrollees 8-14 to maximize the participant usability. Post-hoc analysis to determine the difference in the usability survey response before and after redesign was difficult because of the small cohort and frequent zero response in disagree or neutral categories. We did find the percent of enrollees who responded with agree or strongly to *the overall look and feel of the App is visually appealing* (question 3) increased nonsignificantly from 60% to 100% (*P*=.09).

Enrollees had asthma data collected, either inhaler use or EMA, on 89% of days with a significant increase when comparing enrollees before and after the redesign (86%-92%, *P*=.01). EMA data were obtained on 20% of study days for all enrollees and significantly increased after the redesign (10%-40%, *P*=.006). The redesign increased the data acquisition, and this might represent improvements in the ease of use. No significant differences were observed in the frequency of EMA reports by age (*P*=.48), gender (female 27% and male 13%, *P*=.28), or weekend versus weekday (14% and 22%, respectively, *P*=.09).

**Table 2:** Demographics of partcipants (N=14).

Demographics		Participants, n (%)
**Age in years**		
	8-10	9 (64)
	11-13	3 (21)
	14-16	2 (14)
**Gender**		
	Male	5 (36)
	Female	9 (64)
**Race**		
	White	0 (0)
	Black	13 (93)
	Other	1 (7)
**Ethnicity**		
	Hispanic	0 (0)
	Non-Hispanic	14 (100)
**Insurance**		
	Medicaid	11 (79)
	Private	1 (7)
	Self-pay	2 (14)

**Table 3:** Clinical characteristics and medication adherence of partcipants.

Acute care visits including emergency department, hospital, or intensive care unit	Pre-enrollment (n)	Postenrollment (n)
2 months	4	1
6 months	5	3

### Asthma Symptoms and Contextual Factors Impact on Adherence

EMA reports during the study period frequently included coughing (42/110, 38%), wheezing (8/111, 7%), chest tightness (9/109, 8%), stress (12/109, 11%), not relaxed (43/107, 40%), not happy (51/111, 46%), angry (11/111, 10%), and bored (57/109, 52%). In this population of high-risk youth with asthma, symptoms of wheezing or chest tightness were never reported beyond *a little* with only coughing being infrequently reported as *a lot*. Multimedia Appendix 1 provides a detailed description of responses. A total of 10 new asthma symptoms episodes were detected in the cohort over 2 months.

Our exploratory generalized linear mixed models indicated that the participants’ controller medication adherence might increase on days when asthma symptoms (9.2%; 95% CI 0-18.5) or boredom were reported compared with days when reports demonstrated no asthma symptoms or boredom (Table 1). We investigated the impact of imputing missing report days as no symptom days and found a similar effect size but with a narrower CI (9.9% adherence; 95% CI 4-15). We explored this further and found the adherence was higher on days with reports of nonsymptoms than on nonreport days (35.1% vs 25.2% adherence, *P*<.001), suggesting nonreport days were similar to nonsymptom days but might represent days with even lower adherence. As nonreport days had significantly lower adherence, we excluded them from the analysis of contextual factors that impact the adherence to avoid overestimating the significance of symptoms on the adherence.

### Pre- and Postenrollment Emergency Department and Hospital Visits

One patient experienced an emergency department visit while enrolled in the 2-month study compared with 4 acute care visits in the cohort 2 months pre-enrollment (Table 3). The 4 pre-enrollment acute care visits included one intensive care unit admission. In addition, 1 patient with an emergency department visit during the study period reported asthma symptoms in the days preceding the emergency department visit. During the 6 months pre-enrollment, our study cohort had 5 visits to the acute care setting, including the abovementioned intensive care unit visit compared with 3 acute care visits in the 6 months postenrollment.

## Discussion

### Principal Findings

Smartphone asthma monitoring was feasible and acceptable as reported by children and their families in a population of youth aged 8-16 years with high-risk asthma. The iterative design approach successfully used youth and family engagement to increase data attainment during the study period. Despite being a small-scale pilot trial, the smartphone approach resulted in high-frequency data attainment and began demonstrating statistical associations between reported asthma symptoms and emotional state and participants’ daily adherence patterns. Understanding the reasons for daily adherence behavior among high-risk youth with asthma might lead to better-tailored interventions that maximize the adherence behavior. Nevertheless, larger studies should be performed to determine the generalizability of the findings.

### Limitations

This feasibility trial was designed to test the acceptability and feasibility among patients, including the collection of real-time asthma data. It was not powered to find significant and generalizable differences in the adherence or other clinical outcomes. Additionally, the adherence rate (30%) reported in this trial, which was comparable to other studies, might have been artificially increased because of the Hawthorne Effect. Participants were aware of the medication monitoring and the intervention permitted patients to manually enter medication use when the Bluetooth devices ceased functioning. This approach was adopted as it was preferred by families. One parent noted their child had her inhalers at her grandparents one weekend and was away from the smartphone. Ultimately, the inhaler caps stored this information and the information was captured once in proximity to the smartphone. Thus, larger studies validating youth response to these symptom questions compared with other measures of risk, such as asthma control and emergency department use, are warranted. We cannot confirm the presence or absence of asthma symptoms reported with EMA, but previous studies suggested that this approach is valid in multiple disease states, including asthma [19,20,23]. Furthermore, individual participants may experience symptoms differently because of different body awareness, and this could impact their symptom reporting, particularly their severity of reported symptoms. More so, as a pilot study, we permitted the youth and parents to decide whether the child was to report symptoms alone or in collaboration with the parent. We used interviews to assess how the approach worked for them. Youth would frequently report symptoms alone but had discussions with their parent, which was consistent with the literature suggesting youth value a shared asthma experience with their caregiver and these ages are transitioning to the primary responsibility of asthma care. Unfortunately, we did not have an objective assessment to determine the degree of parental involvement in reporting, but this will be added to a subsequent larger study. Finally, this pilot study was conducted at 2 centers in a high-risk population; therefore, larger studies are needed to determine whether the results are generalizable to other populations.

### Comparison with Prior Work

Asthma symptoms are known to vary over time because of numerous factors, including environmental exposures, medication adherence, physical exertion, and the natural progression of the disease. Mulvaney et al demonstrated that EMAs focused on asthma are associated with the established measures of asthma control (eg, Asthma Control Test) but also noted the difficulty in obtaining patient self-reported medication use [20]. Our study adds to this research by using newer technology for passive collection of medication use data, Bluetooth inhaler devices, and demonstrating an association of contextual factors, such as asthma symptoms and emotional state by EMA, with the adherence device data. The calculated adherence over days, weeks, months, and years is a series of discrete events occurring in patients’ daily life that can be affected by mood and motivation. Research in other chronic diseases suggests that day-to-day factors such as control belief, mood, and social support affect daily adherence, often mediated by daily motivation [17]. Our pilot study furthers this investigation into a population of high-risk youth with asthma and suggests daily asthma symptoms and mood may impact their adherence; this suggests asthma symptoms, if recognized, may be a motivational tool for adherence in this population. Our finding associating boredom with higher adherence deserves further exploration. Boredom is typically considered a negative emotion but may also represent a time when youth are better able to adhere to medications as they are less preoccupied and distracted by other daily activities. Further studies may consider assessments of these and other momentary factors in the home environment among a larger cohort.

Ultimately, the recurrence of new asthma symptom episodes, which we observed 10 times, may be an antecedent to an emergency department or hospital visit. Although this study was not powered to determine the association of recurrent asthma symptoms and emergency department visits, it is noteworthy that our 1 patient with an emergency department visit reported recurrent asthma symptoms the day before their presentation to the acute care setting.

The recruitment process was designed to enroll high-risk patients with asthma to develop a technological tool specific to the unique needs of this population. Our participants were predominantly minority with comorbid diagnosis, insured by Medicaid, and experienced numerous acute care visits before enrollment. During KII conducted postintervention, all participants enrolled in this study indicated they had regular access to smartphones, although children who were at younger ages frequently used their parents’ phones. The near-universal adoption of smartphone technology has the potential to improve access to care for high-risk children with asthma who can be difficult to reach and maintain in primary care practices.

Our technology design process included an iterative approach based on the frequent engagement pretrial and during the clinical trial’s final visit. The redesign elements (eg, 4-digit automated log-in) determined by engagement were not resource-intensive but did result in a marked improvement in data accrual, supporting the importance of patient engagement when designing an mHealth platform [29-31]. The frequent rate of data accrual combined with survey and interview data consistently conveyed ease in the use of this approach. The concept of monitoring symptoms and medication use in real time in a patient’s home environment could result in more timely care delivery based on current symptoms with fewer barriers to care. Ultimately, approaches to integrate technology to improve care processes for chronic disease, once monitoring is successful, are limitless. Huckvale et al noted common weaknesses in many asthma apps that should be considered in the design of future research; these include lack of consistency with best practice guidelines, ability to protect privacy, accounting for local variation in care, and demonstration of proven clinical efficacy [32,33].

### Conclusions

Patients and families reported that using smartphones for real-time asthma monitoring was acceptable. The youth’s daily medication adherence demonstrated a tendency to increase during days with reports of asthma symptoms or boredom. Future research should investigate other contextual factors that may impact changes in daily adherence behavior and approaches to effectively use monitoring with provider-informed interventions to improve adherence behavior and clinical outcomes.

## Appendix

Multimedia Appendix 1 Youth-reported asthma symptoms and emotional state with ecological momentary assessment over smartphones.

Multimedia Appendix 2 Patients and caregiver usability assessed by postintervention survey. SAMS: Smartphone Asthma Monitoring System.

## Abbreviations

EMA: ecological momentary assessment
KII: key informant interview
SAMS: Smartphone Asthma Monitoring System
SMS: short message service
