# Supplementary material for: Smartphones for Real-time Assessment of Adherence Behavior and Symptom Exacerbation for High-Risk Youth with Asthma: Pilot Study
Source: JMIR Pediatr Parent. 2018 Oct 5;1(2):e8. doi: 10.2196/pediatrics.9796 (PMC6716478; doi:10.2196/pediatrics.9796)
Supplement: Multimedia Appendix 1 [file pediatrics_v1i2e8_app1.pdf]

Multimedia Appendix 1. Youth-reported asthma symptoms and emotional state with ecological momentary assessment over smartphones.

Ecological Momentary Assessment				
Asthma Symptoms, n (%)	Not at all	A little	A lot	Extremely
Are you coughing?	68 (61.8)	40 (36.4)	2 (1.8)	0
Are you wheezing?	103 (92.8)	8 (7.2)	0	0
Does your chest feel tight?	100 (91.7)	9 (8.3)	0	0
Emotional State, n (%)				
Are you stressed?	97 (89.0)	11 (10.1)	0	1 (0.9)
Are you angry?	100 (90.1)	7 (6.3)	1 (0.9)	3 (2.7)
Are you bored?	52 (47.7)	48 (44)	3 (2.8)	6 (5.5)
	Extremely	A lot	A little	Not at all
Are you happy?	51 (46)	41 (36.9)	14 (12.6)	5 (4.5)
Are you relaxed?	43 (39.5)	35 (32.1)	20 (18.4)	11 (10.1)
