# Supplementary figures and images for: Smartphones for Real-time Assessment of Adherence Behavior and Symptom Exacerbation for High-Risk Youth with Asthma: Pilot Study
Source: JMIR Pediatr Parent. 2018 Oct 5;1(2):e8. doi: 10.2196/pediatrics.9796 (PMC6716478; doi:10.2196/pediatrics.9796)

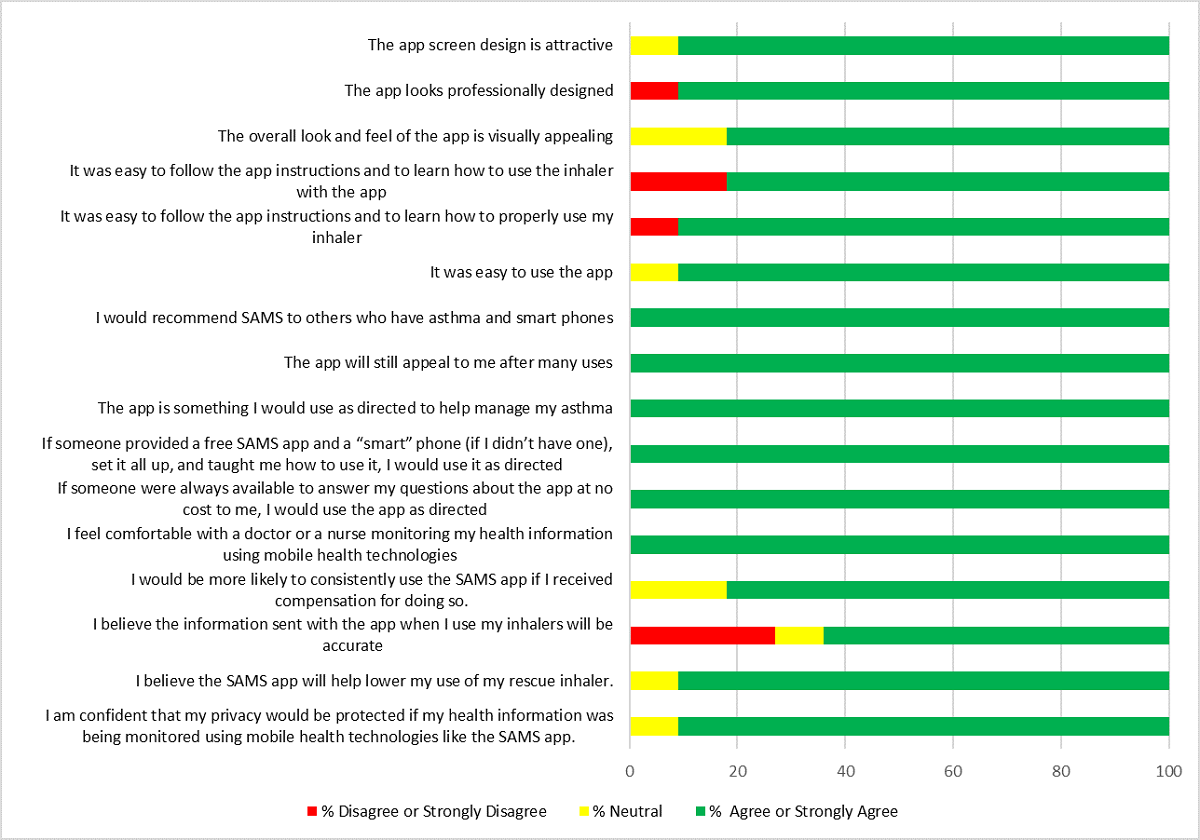

Supplement: Multimedia Appendix 2 [file pediatrics_v1i2e8_app2.png]
